# Internal medicine in South Africa: A historical and contemporary overview

**DOI:** 10.4102/jcmsa.v1i1.39

**Published:** 2023-11-30

**Authors:** Somasundram Pillay

**Affiliations:** 1 Department of Internal Medicine, Faculty of Medicine, University of KwaZulu-Natal, Durban, South Africa

South Africa’s healthcare milieu has evolved dramatically, with the discipline of internal medicine standing at the crossroads of this transformative journey. This editorial offers an in-depth exploration of the discipline’s genesis, some of its pioneers, significant milestones, global interplays, current challenges, and potential future trajectories.

## Genesis and formative years

Internal medicine’s inception in South Africa can be traced back to the waning years of the 19th century, when the nation laid the groundwork for its first medical education institutions. As the 20th century unfolded, pivotal bodies such as the College of Medicine of South Africa emerged. This institution played a crucial role in delineating the postgraduate medical education landscape and bestowing a definitive stature upon the discipline of internal medicine.^1^

## Pioneers and milestones

The progress of internal medicine in South Africa is a tapestry interwoven with the contributions of pioneering figures. These luminaries championed the cause of medical excellence, research, and equitable patient care. Their endeavours were complemented by significant milestones, including groundbreaking research, the establishment of specialised care centres, and the formulation of guidelines tailored to the nation’s unique health challenges.

Highlighting pioneering figures specifically from the realm of internal medicine in South Africa can be a challenging endeavour because of the myriad professionals who have contributed immensely over the years. However, a few figures and entities have made significant contributions to the field and can be observed as pioneers:

- **Professor Bongani Mayosi:** Recognised as a cardiologist, Prof. Mayosi’s contributions to conditions such as rheumatic heart disease have been monumental. His leadership roles, especially as the Dean of the Faculty of Health Sciences at the University of Cape Town, have been crucial in shaping medical education and research in the region.
- **Professor Solomon R. Benatar:** Prof. Benatar’s work in internal medicine, especially regarding the ethics of global health and human rights, has been of great significance.
- **Dr Motasim Badri:** Renowned for his epidemiological work on HIV and AIDS, Dr Badri’s contributions have been vital for internal medicine given South Africa’s significant HIV and AIDS burden.
- **The College of Physicians of South Africa:** This institution has been a linchpin in shaping internal medicine in South Africa, with its associated professionals setting crucial standards and advancing the speciality.
- **Professor Ntobeko Ntusi:** As the Chair and Head of the Department of Medicine at the University of Cape Town and Groote Schuur Hospital, Prof. Ntusi is renowned for his work in cardiology, especially in the areas of inflammatory heart diseases and cardiac imaging.
- **Professor Keertan Dheda:** A respiratory physician and researcher, Prof. Dheda has made significant contributions to understanding tuberculosis, a disease of particular concern in South Africa. His work has informed both clinical practice and public health policies.

## Milestones in research and care

- **Tuberculosis (TB) research:** South Africa has been at the forefront of TB research, given the high prevalence of the disease. Groundbreaking research into multidrug resistant TB, spearheaded by institutions such as the University of Cape Town and Stellenbosch University, has led to better diagnostic methods and treatment protocols.^2,3^
- **HIV and AIDS treatment and research:** South Africa has the largest antiretroviral treatment programme globally. Landmark studies, such as those conducted by the Centre for the AIDS Programme of Research in South Africa (CAPRISA) Consortium, have illuminated the pathogenesis of HIV and have paved the way for innovative treatments and preventative measures.^4^

The tapestry of internal medicine in South Africa is rich and varied, thanks to the pioneering figures who paved the way and the significant milestones that have shaped the discipline. As we look back at the legacy, it is clear that the fusion of dedicated individuals and groundbreaking achievements has been instrumental in advancing the field.

## Apartheid’s shadow

The apartheid era, spanning from 1948 to 1994, was a tumultuous period that brought its unique set of challenges to the medical realm. The policies of racial segregation deeply affected medical education and practice. Physicians of African descent faced myriad hurdles, from limited educational opportunities to curtailed professional trajectories in predominantly white institutions.^5^ Yet, in the face of adversity, the resilience of the medical community was evident. Many, transcending racial lines, championed the cause of equitable healthcare, challenging the divisive policies of the apartheid administration.^6^

## Post-apartheid renaissance

With the fall of apartheid came a reinvigorated zeal for internal medicine. The dismantling of racial impediments catalysed efforts to heal the scars of past disparities. The inception of a comprehensive national health system underscored the nation’s commitment to ensuring every citizen’s access to top-tier healthcare.^7^

## Current landscape and global interactions

Navigating the 21st century, South Africa grapples with an intricate health challenge mosaic, ranging from the dual epidemic of HIV and AIDS and tuberculosis to rising non-communicable diseases and maternal-infant health issues.^8^ In response, internal medicine has displayed remarkable versatility. The discipline’s evolution is characterised by innovative clinical establishments, a rejuvenated focus on research, and a symbiotic amalgamation of traditional and modern medical paradigms.^9^ Furthermore, South Africa’s internal medicine trajectory is interlinked with global trends, benefiting from international collaborations, shared research, and global health initiatives.

## Prospects and horizons

The legacy of internal medicine in South Africa is a testament to the nation’s broader historical canvas, characterised by adversities, resilience, and transformative endeavours. As the discipline strides forward, it remains emblematic of South Africa’s unwavering commitment to a healthcare paradigm that is inclusive, equitable, and progressive.
